# Molecular Events Linking Oxidative Stress and Inflammation to Insulin Resistance and *β*-Cell Dysfunction

**DOI:** 10.1155/2015/181643

**Published:** 2015-07-14

**Authors:** Kevin Noel Keane, Vinicius Fernandes Cruzat, Rodrigo Carlessi, Paulo Ivo Homem de Bittencourt, Philip Newsholme

**Affiliations:** ^1^School of Biomedical Sciences, Curtin Health Innovation Research Institute, Biosciences, Curtin University, 6102 Perth, WA, Australia; ^2^Department of Physiology and Biophysics, Institute of Biomedical Sciences, University of São Paulo, São Paulo, SP, Brazil; ^3^Post-Graduate Program in Medical Sciences: Endocrinology, Federal University of Rio Grande do Sul, 90035-003 Porto Alegre, RS, Brazil; ^4^Department of Physiology, Institute of Basic Health Sciences, Federal University of Rio Grande do Sul, 90050-170 Porto Alegre, RS, Brazil

## Abstract

The prevalence of diabetes mellitus (DM) is increasing worldwide, a consequence of the alarming rise in obesity and metabolic syndrome (MetS). Oxidative stress and inflammation are key physiological and pathological events linking obesity, insulin resistance, and the progression of type 2 DM (T2DM). Unresolved inflammation alongside a “glucolipotoxic” environment of the pancreatic islets, in insulin resistant pathologies, enhances the infiltration of immune cells which through secretory activity cause dysfunction of insulin-secreting *β*-cells and ultimately cell death. Recent molecular investigations have revealed that mechanisms responsible for insulin resistance associated with T2DM are detected in conditions such as obesity and MetS, including impaired insulin receptor (IR) signalling in insulin responsive tissues, oxidative stress, and endoplasmic reticulum (ER) stress. The aim of the present review is to describe the evidence linking oxidative stress and inflammation with impairment of insulin secretion and action, which result in the progression of T2DM and other conditions associated with metabolic dysregulation.

## 1. Introduction

The obesity epidemic and the consequent development of metabolic syndrome (MetS) are an increasingly significant health problem, associated with reduced life span and quality of life. Insalubrious diet habits, physical inactivity, urbanisation, genetic predisposition, and aging contribute to the higher prevalence of these diseases across populations worldwide. MetS is a term given to a condition associated with the collection of a series of risk predictors that are associated with metabolic diseases including obesity, cardiovascular disease, diabetes mellitus (DM), and more recently dementia [1–3]. These risk factors include changes in plasma triglycerides, low density lipoprotein (LDL) to high density lipoprotein (HDL) cholesterol ratio, high fasting glycaemia, and alterations in blood pressure and adiposity [1, 2]. Aberrant lipid and glucose metabolic turnover, and decreased action or subsequent dysfunctional release of insulin, is central to the development and progression of diabetes, particularly in relation to the onset of insulin resistance which is associated with impaired insulin signalling in insulin sensitive target tissues including muscle, liver, and adipose tissue.

Oxidative stress and inflammation are key players in insulin resistance progression and the establishment of type 2 DM (T2DM). As obesity is established and body weight increases with age, a parallel state of chronic inflammation, characterised by an elevation of proinflammatory cytokines, can induce changes and switch the metabolic homeostatic set points, leading to T2DM [4]. In response to inflammatory stimuli, an increased migration and infiltration of macrophages may occur in peripheral tissues, including pancreatic islets, liver, and adipose tissue, so that cell and tissue malfunction results including reductions in insulin secretion [5–7].

Elevated energy intake and/or low expenditure (insufficient physical activity) leads to abnormal nutrient metabolism that culminates in the accumulation of intracellular and extracellular lipids contributing to lipotoxicity, a process widely reported for T2DM. Notably, increasing apolipoprotein E (ApoE) and related isoforms (E4, E3, and E2) have been correlated with increasing body mass [8] and specifically ApoE4 was associated with increasing levels of serum insulin and glucose [9–11]. Furthermore, it has been demonstrated that mice deficient in ApoE4 had reduced lipid deposition in the liver and adipose tissue, which eloquently connects dysfunctional lipid metabolism to obesity [12]. Additionally, the formation and accumulation of ceramide, an important lipid metabolite, have also been associated with several chronic diseases, including cardiovascular disease, obesity, and T2DM [13]. Interestingly, it has recently been shown that ceramide accumulation may impact energy balance through induction of lipotoxicity and ER stress in the hypothalamus [14]. Continuous overnutrition (i.e., high glucose and lipid diets) leads to glucotoxic and lipotoxic effects or, in combination over a chronic period, glucolipotoxic conditions. Importantly, glucolipotoxicity affects mitochondrial function including impairment of electron transport chain activity, leading to increased reactive oxygen species (ROS) production which, in turn, triggers inflammation in both peripheral tissues and insulin secreting *β*-cells. Furthermore, excess nutrients, such as abnormally high glucose and fatty acids, promote endoplasmic reticulum (ER) stress, which reduces the ability of *β*-cell to efficiently secrete insulin, contributing to the increased inflammation, oxidative stress, and elevated circulating nutrients including glucose. Mechanistically, excessive generation of ROS can affect DNA, protein, and lipid integrity and consequently lead to cell death. Although it is still difficult to attribute causal effects of oxidative stress and inflammation in relation to each other in DM, both play a significant role and mediate the progression of DM. As much as novel molecular approaches have revealed several different pathways and targets of insulin secretion and action during progression of T2DM, in this paper we examine the commonalities and differences in metabolic and molecular processes that emanate from the extrapancreatic periphery towards the pancreatic *β*-cell leading to the dysfunctions that characterise T2DM.

## 2. Insulin Resistance

Obesity and MetS are correlated metabolic diseases associated with chronic low-grade inflammation. The inflammatory response is well characterised as an adaptive and protective process for multicellular organisms to oppose infections, and it also permits tissue repair [15]. On the other hand, in conditions such as obesity, MetS, and T2DM, chronic inflammation induces changes in metabolic function and alters homoeostatic set points, which exacerbate the disease. For instance, the link between the metabolic disturbances, such as high lipid profile and development of atherosclerosis, was first demonstrated in 1973 and classified as a catastrophic disease [16]. However, the relationship between obesity and T2DM was only illustrated* in vivo* twenty years later, in 1993 [17]. Here, it was reported that adipose-derived tumour necrosis factor alpha (TNF-*α*) levels in mice were increased during the advancement of obesity, but when TNF-*α* was neutralised, insulin sensitivity was improved [17].

Later, it was found that the mechanism by which TNF-*α* suppressed insulin receptor (IR) activity involved serine phosphorylation of IRS1 (insulin receptor substrate-1), an important signalling protein immediately downstream of the IR [18]. TNF-*α* attenuated insulin-mediated modifications of cell function and metabolism, demonstrating the importance of this cytokine in linking adipose tissue inflammation with insulin resistance. In normal conditions, binding of insulin to the IR induces the production of triacylglycerols from diet-derived fatty acids and glucose-derived glycerol 3-phosphate. Therefore, insulin promotes a simultaneous uptake of lipids and glucose into adipose tissue* in vivo*. Any impairment of these interactions will lead to a surplus of circulating glucose and fatty acids, which is predominantly observed in T2DM. Conversely, in periods of fasting, starvation, and strenuous exercise, adipose tissue will release nonesterified fatty acids (NEFAs) and glycerol into the vasculature, under the action of various lipases to replenish the plasma nutrient levels and spare glucose for brain function in these conditions.

The adipose tissue is also able to synthesise and releases adipokines into the circulation, including leptin and adiponectin. Leptin was demonstrated to modulate satiety and energy balance, an effect dependent on neuroendocrine signalling in the hypothalamus [19]. Similarly, adiponectin was shown to promote insulin sensitivity, and mice with adiponectin-deficiency are highly insulin resistant [20]. In addition, elevated basal adiponectin levels may be associated with a reduced risk of T2DM [21]. Therefore, adipokines are considered to modulate insulin sensitivity in the key insulin target organs, including liver and skeletal muscle. However, in chronic expansion of adipose tissue, low-grade inflammation occurs with consequent infiltration of immune cells that can lead to a reduction in adipokine secretion, subsequently resulting in systemic insulin resistance.

### 2.1. The Role of Unresolved Inflammation in Extrapancreatic Periphery

Macrophage accumulation in obese adipose tissue is common, and here they secrete proinflammatory cytokines that modulate adipose tissue glucose and lipid metabolism [20]. Tateya et al. demonstrated that F4/80^+^ CD11b^+^ macrophages were present in adipose tissue of lean mice and represented 5% of the stromal vascular fraction. However, this figure may be increased by up to 30% in adipose tissue of obese mice [20]. The infiltration of these immune cells into adipose is significant as macrophage-derived TNF-*α* and interleukin-6 (IL-6) can impair lipoprotein lipase activity and thus may increase blood triacylglycerol concentration. Furthermore, TNF-*α* can promote hormone-sensitive lipase activity in adipose tissue, which may result in release of NEFA into the blood, while concomitantly reducing insulin-stimulated glucose uptake via impaired insulin signalling as outlined above. Consequently, these effects would encourage increased plasma lipid levels, against the backdrop of reduced lipid disposal by adipose tissue, which perpetuates lipotoxicity in the T2DM condition.

Increasing plasma concentrations of NEFA and ceramide are important in connecting nutrient metabolism with inflammation. Accordingly, ceramide was shown to induce IL-1*β* secretion from macrophages in obese individuals and high-fat diet (HDF) fed animals [22], while at a mechanistic level, NEFAs activated the NOD-like receptor family and pyrin domain containing 3 (NLRP3) inflammasome in haematopoietic cells and promoted insulin resistance [23]. A recent key publication revealed that activation of the macrophage inflammasome using islet amyloid polypeptide (IAPP) was dependent on both glucose and fatty acid metabolism [24], leading to subsequent production of inflammatory cytokines IL-1*β* and IL-18. A follow-up study demonstrated that both glucose and minimally modified low density lipoprotein (mmLDL), both of which are elevated in T2DM [25], were required for full IAPP-mediated activation of NLRP3 inflammasomes in bone marrow-derived macrophages. Furthermore, Toll-Like Receptor-4 (TLR4) downstream pathways were found to be critical for transducing these signals [24].

### 2.2. The Central Role of Infiltrating Macrophages

The activation status of infiltrating macrophages is important in the progression of metabolic diseases. Two different polarisation states, M1 (proinflammatory) and M2 (anti-inflammatory), have been characterised so far. The proinflammatory M1 form is stimulated by proinflammatory mediators such as lipopolysaccharide (LPS), TNF-*α*, and interferon-*γ* (IFN-*γ*). These macrophages produce and secrete TNF-*α*, IL-1, and IL-6, enhancing the inflammatory response. Conversely, the M2 anti-inflammatory phenotype has significantly reduced proinflammatory characteristics, and these cells release high levels of anti-inflammatory cytokines, for instance, IL-10. Interestingly, ingestion of a diet high in lipid content was shown to polarise Kupffer cells of the liver towards the M1 phenotype [26]. These cells are resident macrophages of the liver and this polarisation was associated with the pathogenesis of obesity-induced insulin resistance and fatty liver disease [26].

In addition, TNF-*α* production by M1 macrophages in the liver can promote increased hepatic glucose output via gluconeogenesis and by decreasing glycogen content, while simultaneously enhancing lipid production and storage through inhibition of intracellular lipases and providing intracellular fatty acids for triacylglycerol synthesis. Thus, elevated TNF-*α* in the obese liver may increase blood glucose levels and promote fatty liver disease [26]. However, there is a heterogeneous population of immune cells in the liver, but Kupffer cells, in particular, are believed to facilitate both insulin resistance and hepatic steatosis and steatohepatitis, which are associated with increased c-Jun N-terminal protein kinase (JNK1) activation and consequent lowering of heat shock protein (HSP) pathways, which are anti-inflammatory [27]. Interestingly, chemical removal of these cells can improve insulin sensitivity during consumption of a high-fat diet. Therefore, the delicate balance and adaptability of macrophages between M1 and M2 phenotypes are important to liver metabolism. Consequently, maintenance of the M2 phenotype over M1 phenotype is desirable in the liver and key for appropriate glucose and lipid production along with subsequent release.

Taken together, these data suggested that the high nutrient milieu observed in T2DM may activate circulating macrophages that could possibly lead to chronic low-grade inflammation, which is a hallmark of obesity and T2DM. Moreover, interactions of macrophages and production of proinflammatory cytokines can negatively affect metabolic processes in tissues that are physiological targets for insulin. These inflammatory exchanges may lead to hyperglycaemia and dyslipidaemia, which are important characteristics indicative of obesity, T2DM, and MetS.

### 2.3. Impaired Insulin-Signalling Pathways

Insulin resistance does play a key role in the pathogenesis and progression of chronic metabolic diseases that are proinflammatory in nature, such as obesity, T2DM, brain dysfunction, and heart disease [28]. Insulin resistance is an important health issue since it flourishes silently much before the onset of such metabolic manifestations [15, 29]. Insulin resistance refers to impaired or failed intracellular transduction of the insulin-mediated signalling cascade in sensitive tissues, especially the liver, skeletal muscle, and adipose tissue. This leads to an impaired disposal of blood glucose along with an elevated hepatic glucose output, both combining to result in elevated plasma glucose. High levels of glucose promote an increased demand on pancreatic *β*-cells to synthesise and secrete insulin so as to reestablish appropriate blood glucose levels. Since this compensatory mechanism may reduce glucose levels during prediabetes and early stages of DM development, chronic and persistent insulin resistance exposes the *β*-cells to an excess of glucose and lipids, thereby promoting *β*-cell dysfunction, failure, and death. However, insulin sensitivity can be improved using pharmacological drugs, control of diet, and regular exercise [30]. To investigate the mechanisms leading to insulin resistance, one must first understand insulin signalling in the context of normal insulin-mediated interactions that are observed in nondiabetic models.

#### 2.3.1. Disruption of Insulin Receptor (IR) and Insulin Receptor Substrate (IRS)

Insulin is released into the vasculature by *β*-cells in response to elevated blood glucose levels, following ingestion of food. Insulin elicits its anabolic effects via association with the transmembrane IR, present in target tissues. These key membrane-bound receptors are present in cells that store surplus carbohydrate in the form of glycogen (liver and muscle) or as triacylglycerol (adipose tissue). The IR is composed of four peptide subunits (two *α*-chain subunits and two *β*-chain subunits) and is a heterotetrameric tyrosine kinase receptor. The interaction with insulin induces autophosphorylation of the receptor at tyrosine residues (Tyr^1158^, Tyr^1162^, and Tyr^1163^) [31], and this initiates the recruitment and phosphorylation of the intracellular adapter proteins (IRS).

Thirteen different IRS isoforms have been described, but isoforms 1 and 2 have been studied extensively since they are widely distributed among different cell types and are mainly activated in skeletal muscle [32], which is responsible for approximately 75% of insulin-stimulated blood glucose uptake in the body [32, 33]. Phosphorylated IRS1, to a lesser extent IRS2, induces activation of the PI3K lipid kinase via binding with the p85 regulatory subunit of PI3K. Activated PI3K converts phosphatidylinositol 3,4-bisphosphate (PIP2) to PI(3,4)P2 and phosphatidylinositol 3,4,5 triphosphate (PIP3), via the p110 catalytic subunit. This conversion activates 3-phosphoinositide-dependent protein kinase 1 (PDK1) that subsequently recruits and phosphorylates protein kinase B (pAkt) at the plasma membrane. PDK1 can also activate atypical protein kinase C (aPKC) which also regulates glucose metabolism [34]. Downstream of these interactions, pAkt has over 100 substrates that regulate many cellular processes including cell proliferation, differentiation, endocytosis, survival, and glucose homeostasis [35]. Three isoforms of Akt exist, and Akt2 is recognised as the most abundant in insulin sensitive tissues. Interestingly, when Akt2 was deleted in knockout mice, increased insulin resistance was observed illustrating the important physiological role played by Akt2 in mediating glucose homeostasis [36]. Mechanistically, Akt is an important regulator of translocation of GLUT4 vesicles to the plasma membrane, which is critical for the intracellular uptake of free glucose in insulin sensitive tissues [37, 38].

**Figure 1:**
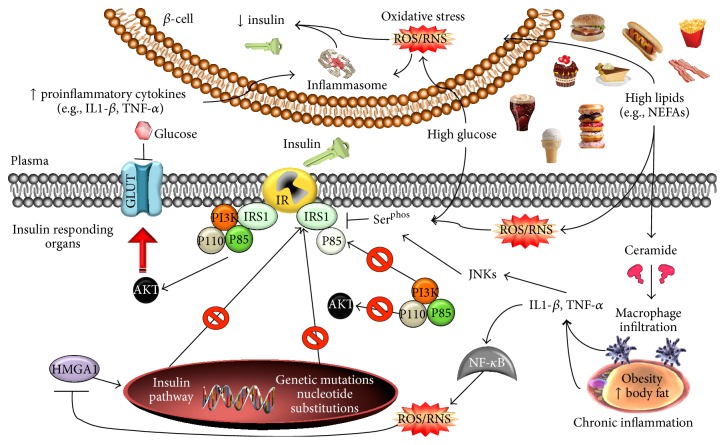
Insulin resistance and the role of inflammation. Overnutrition leads to high levels of lipids and glucose and overtime development of obesity and metabolic syndrome (MetS), ultimately causing chronic low-grade inflammation. High nutrients can modulate insulin resistance by altering the insulin-signalling cascade through changes in IRS1, PI3K, and AKT phosphorylation. High lipids can also promote inflammation through generation of ceramide, and high glucose increases overall oxidative stress. During T2DM progression the insulin resistant tissues promote the exhaustion of insulin secreting *β*-cells, which activates defensive mechanisms leading to lower insulin release.

Appropriate insulin signalling may be interrupted because of either genetic alterations or physical changes to any of the aforementioned signalling nodes, and this may manifest as insulin resistance. Mutations and serine hyperphosphorylation of IRS proteins are especially associated with development of insulin resistance, as they are thought to decrease the interaction of IRS with PI3K (reviewed by [39]). Previously, homozygous disruption of IRS1 transcription led to mild insulin resistance [40], while IRS2-knockout mice exhibited severe insulin resistance [41]. Furthermore, in T2DM patients, many precise amino acid substitutions in IRS1 proteins are believed to alter protein function, but some of these substitutions have been controversial. For example, researchers have reported that Gly972Arg is a common polymorphism in T2DM patients [42, 43], but others have failed to observe similar findings in other T2DM populations [44]. In addition, hyperphosphorylation of serine at residues Ser^302^, Ser^307^, Ser^612^, and Ser^632^ in IRS1 was suggested to be responsible for increased insulin resistance in animal models [39]. In fact, uncontrolled proinflammatory cytokines synthesis and secretion, and activation of proinflammatory signalling proteins activation, such as tumour necrosis factor alpha (TNF-*α*) and the isoform 1 of c-Jun N-terminal protein kinase (JNK1) in response to adipose tissue expansion, can induce serine hyperphosphorylation in IRS1 [18, 45], especially at residue Ser^636^ (Figure 1). However, it is not entirely known which specific serine residues or combination thereof require hyperphosphorylation to elicit the insulin-resistant phenotype, as excessive phosphorylation at Ser^337^ and Ser^636^ has been demonstrated in muscle samples from patients with metabolic syndrome, but not at Ser^307^, Ser^789^, or Ser^1101^ as reported by others [45]. Moreover, serine hyperphosphorylation at residue Ser^312^ marks IRS1/2 for degradation, which dampens the IR-mediated signalling relay. Taken together, these data demonstrate the complexity of the role played by IRS proteins, but also their importance in modulating insulin resistance.

#### 2.3.2. Disruption of PI3K Function

Dysregulation of PI3K activity is another molecular mechanism that may attenuate insulin signal transduction. PI3K lipid kinases are composed of two polypeptide subunits, one p110 catalytic subunit and a p85, p65, or p55 regulatory subunit, and these proteins are classified according to the combination of both domains. Importantly, a balance between the active heterodimer and the individually expressed inactive regulatory domains exists in the cytosol. This system allows for tight regulation and appropriate activation of the PI3K-Akt pathway, where the regulatory domains compete for IRS binding sites with the active heterodimer [39]. However, it was suggested that excessive expression of individual regulatory domains (e.g., p85) in skeletal muscle was responsible for blocking the binding of the signalling heterodimer with IRS in pregnancy-induced insulin resistance [46]. Conversely, in mice with the genetic deletion of p85 in the liver, improved hepatic and peripheral insulin sensitivity was shown [47], which eloquently demonstrates the impact of dysfunctional PI3K-signalling on insulin resistance.

Alternatively, decreased IR expression or desensitisation to the insulin ligand may occur and may be involved in the insulin resistant phenotype. The precise molecular mechanisms are not fully understood, but some studies have shown that chronic hyperglycaemia and prolonged hyperinsulinaemia, together with increased ROS/RNS levels, may affect IR gene expression via dysfunction of key transcription factors such as high mobility group AT-hook 1 (HMGA-1) [48]. Importantly, under normal conditions these signalling pathways are regulated by a negative-feedback mechanism to control IR sensitivity [49]. Hyperinsulinaemia is a key pathological characteristic of insulin resistance but it is not clear whether this is a cause or a consequence of insulin resistance. Chronically elevated insulin levels can reduce IR expression in the liver [50, 51] and primary adipocytes [49] and in the kidney [52], and it is a possible mechanism that exasperates development of insulin resistance. In addition, it was suggested that decreased IR expression in the kidney of insulin resistant rats increased sodium reabsorption in the proximal tubule [52], and this salt retention promoted hypertension, which is closely associated with cardiovascular disease, obesity, diabetes, and insulin resistance.

#### 2.3.3. Hyperglycaemia Negatively Impacts Insulin-Responsive Tissues

Interestingly, in skeletal muscle excessively increased carbohydrate levels can also decrease insulin association with the IR and cause decreased IR expression [53]. Supporting experiments that mimicked the milieu of T2DM showed that high glucose and high insulin in combination reduced insulin binding to the IR in adipocytes [54]. These* ex vivo* treatments also decreased the expression of IRS1 and IRS2, further demonstrating inhibition of insulin signal transduction, while these treatments also impacted negatively Akt sensitivity. This latter work revealed the effects of high glucose and insulin on inducing postreceptor defects. However, the precise molecular processes by which elevated carbohydrates promote insulin resistance are not fully understood but are believed to involve modifications of postreceptor molecules such as IRS, PI3K, and Akt.

For instance, increased production of ROS/RNS or decreased antioxidant capacity as a result of increased carbohydrate metabolism in the periphery may modify phosphorylation of these signalling proteins and promote deactivation. In adipocyte and muscle cell lines, it was observed that hydrogen peroxide (H_2_O_2_) reduced insulin signalling and consequently glucose transport [38] (Figure 1). H_2_O_2_ may induce Ser^307^ phosphorylation in IRS1, leading to reduced IRS1 protein expression and increased IRS proteolysis [55, 56]. In addition to oxidative stress, proinflammatory cytokines, including TNF-*α*, can also induce Ser^307^ phosphorylation and thereby decrease the interaction of IRS1 and the IR, impairing signal transduction in a similar manner. Thus, it is not a coincidence that, in obesity and T2DM patients, proinflammatory cytokines and plasma nutrients are chronically elevated, compared to healthy and lean subjects [29, 57]. When these processes are coupled with elevated oxidative stress, a proinflammatory environment is maintained, which leads to the unresolved inflammation and chronic activation of proinflammatory signalling pathways activation (e.g., nuclear factor kappa B (NF-*κ*B) and JNKs). As a consequence, the progression of the disease occurs along with time (several years), and immune cell recruitment occurs in insulin target tissues, such as liver and adipose tissue, but also in tissues not directly associated with insulin action, including the islet [5].

## 3. ***β***-Cell Dysfunction in Diabetes

**Figure 2:**
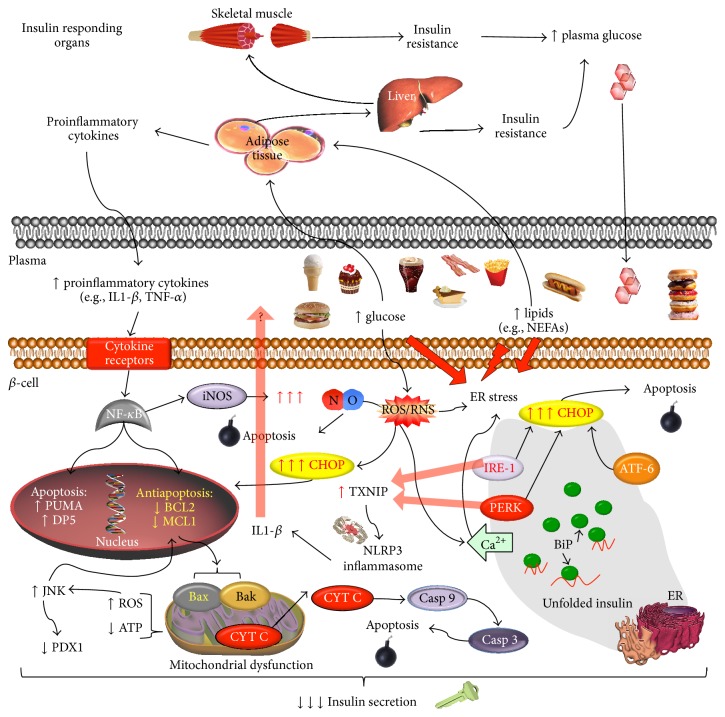
*β*-cell dysfunction in diabetes. Excessive glucose levels lead to high insulin production in *β*-cells. Increased insulin synthesis promotes endoplasmic reticulum (ER) overload, unfolded protein response (UPR), and ER stress. Prolonged ER stress leads to apoptosis and IL-1*β* release through inflammasome activation. Local proinflammatory cytokines induce NF*κ*B activation which promotes proapoptotic gene expression changes. These changes in gene expression favour Bax/Bak oligomerization and mitochondrial outer membrane permeabilization (MOMP), consequently leading to apoptosis. In addition, NF*κ*B-dependent iNOS activation triggers ER stress and apoptosis as well. Lipotoxicity promoted by NEFAs also leads to *β*-cells ER stress via different mechanisms, including reactive oxygen and nitrogen species (ROS/RNS), calcium depletion (Ca^2+^), and ER to Golgi protein traffic impairment.

The major function of the pancreatic *β*-cell is to couple nutrient sensing and subsequent metabolism to insulin release. Therefore, acute exposure of *β*-cells to nutrients, such as glucose, lipids, and some amino acids, promotes insulin secretion. However, incessant exposure to elevated glucose and lipids is known to have detrimental effects on pancreatic *β*-cell insulin secretion [58] and is correlated with onset of insulin resistance in the periphery [59]. These elevated nutrient levels can initially result in enhanced metabolism of *β*-cells and subsequent hyperinsulinaemia, but chronic exposure induces oxidative stress that may cause *β*-cell dysfunction and even *β*-cell death [60]. Consequently, decreased insulin output from this process due to *β*-cell death compounds glucolipotoxicity and initiates the deleterious vicious cycle observed in T2DM (Figure 2).

### 3.1. Oxidative and Nitrosative Stress: A Role in Glucolipotoxicity

Emerging evidence has suggested that the responsiveness of pancreatic *β*-cells to glucose, the major insulin secretagogue, is dependent on the acute regulation of intracellular and, in certain cases, extracellular ROS/RNS [61, 62]. Increased glycolytic flux enhances oxidative phosphorylation and ATP generation, with the concomitant elevation of superoxide (O_2_^•^) anion release from the electron transport chain [63]. Moreover, through the pentose phosphate pathway the conversion of glucose to pentose is an initial response to deviate glucose carbon away from excessive glycolysis and oxidative phosphorylation. However, this pathway can also produce NADPH which via NADPH Oxidase (NOX) activity leads to increased O_2_^•−^ and subsequently H_2_O_2_ formation in the *β*-cell [60]. Persistently high glucose can lead to the generation of ROS via other mechanisms including glucose autoxidation and generation of advanced glycated end products (AGE), which exasperates intracellular ROS levels [64].

Superoxide formation is considered the first stage in a ROS-generating cascade that produces other forms of ROS/RNS [61]. Superoxide can be converted into the less reactive but longer lived species H_2_O_2_, via superoxide dismutase (SOD), which can subsequently generate the highly reactive hydroxyl free radical (OH^•^) by the iron-catalysed Fenton reaction [61, 63, 65]. Furthermore, the reaction of O_2_^•−^ with nitric oxide (NO) or the reaction of H_2_O_2_ with nitrite forms the highly reactive nitrogen free radical, peroxynitrite (ONOO^−^) [61], which induced significant cellular damage in rat and human islets* in vitro* [66, 67] and has been found to be elevated in the islets of NOD mice [68]. For instance, the function of antioxidants in *β*-cells involves multiple steps until the complete reduction to H_2_O_2_. ROS/RNS promote oxidative damage to DNA, proteins, and lipids through nitration, carbonylation, peroxidation, and nitrosylation modifications. These molecular alterations may affect enzyme activity, ion channel transport, or receptor signal transduction and consequently dysregulate gene expression and induce apoptosis [61]. It is well established that ROS-mediated activation of JNK signalling leads to decreased insulin secretion via nucleocytoplasmic translocation of pancreatic and duodenal homeobox-1 (PDX-1), a key transcription factor that binds to the insulin promoter and induces insulin expression [69].

ROS/RNS may also play a dual role in regulating insulin secretion, where excess is detrimental but lower levels aid insulin release. It appears that low levels are required to act as a second messenger for glucose stimulated insulin secretion (GSIS). NO was shown to regulate the interaction between glucokinase and insulin secretory granules [70] and also enhanced granule exocytosis through S-nitrosylation of syntaxin 4, a protein involved in vesicle-plasma membrane docking [71]. Additionally, low H_2_O_2_ levels were suggested to positively regulate mitochondrial Ca^2+^ influx [72], a process that is important for enhancing tricarboxylic acid cycle activity, which is a major driver of the second phase of insulin release [73]. The specific downstream targets of ROS/RNS that positively and/or negatively regulate insulin secretion are still unclear, but possible candidates include various tyrosine kinase receptors, transcription factors, and other protein kinases/phosphatases [62]. Consequently, ROS/RNS may additionally regulate important cell signalling pathways such as PI3K/Akt, mitogen-activated protein kinases (MAPK), JNK, and NF-*κ*B that govern cell proliferation, inflammation, and cell survival.

### 3.2. Antioxidant Systems

Pancreatic *β*-cells have a high metabolic activity but are vulnerable to oxidative stress as they possess reduced antioxidant levels including glutathione peroxidase (GPx) and catalase [61, 65], although they do express other antioxidants including glutaredoxin and thioredoxin [61]. However, oxidative stress occurs in *β*-cells as a consequence of either excessive ROS/RNS production or a failure of antioxidant species to effectively neutralise increasing ROS/RNS levels. For instance, overexpression of SOD sensitised *β*-cells to cytokine-mediated apoptosis [74], while enhanced expression of GPx protected islet *β*-cells from ribose-induced intracellular peroxide production [75]. In addition, antioxidant supplements such as taurine and tempol [76], as well as amino acids with antioxidant properties (e.g., glutamine and arginine) [77, 78], were also demonstrated to protect *β*-cells.

One of the most important nonenzymatic cellular antioxidants is glutathione (GSH, L-*γ*-glutamyl-L-cysteinylglycine), which is also a cofactor for GPx activity. GSH is a tripeptide that provides protection from oxidative stress and inflammation [79, 80], but the* de novo* synthesis is dependent on uptake and utilisation of the amino acid glutamine [81]. Glutamine is the most abundant amino acid in the circulation and is regarded as an important metabolic mediator of insulin secretion [77, 82, 83]. However, levels were suggested to be decreased in T2DM patients [84], as well as other intermediary amino acids and related products, including nitric oxide metabolites [57]. Glutamine is also important modulator of cytoprotective proteins, such the Heat Shock Protein (HSP), which are critical following an oxidative insult or increased endoplasmic reticulum (ER) stress [77, 85]. HSPs are a large family of related proteins that act as molecular chaperones which correctly fold or refold proteins damaged by oxidative processes or the ubiquitin-proteasome system (UPS) [86]. When HSPs are expressed intracellularly they are considered cytoprotective, and recent work has demonstrated that HSPs like HSP27, HSP70, and HSP90 reduced oxidative stress and inflammation [81, 85, 87]. Conversely, when added exogenously, we found that HSP72 reduced *β*-cell insulin secretion, altered cellular bioenergetics, and induced apoptosis* in vitro* [88]. We speculate that the pathogenic extracellular release of HSP70 may occur in the diabetic setting through muscle wasting, through adipose expansion, or via necrotic destruction of neighbouring *β*-cells. However, the precise clinical impact and the molecular mechanisms leading to these proapoptotic effects are currently still under investigation by the authors.

Excessive oxidative and nitrosative stresses also stimulate the expression of heme oxygenase-1 (HO1), which is also known as HSP32 [59], as HO1 is a member of heat shock protein family. HO1 degrades prooxidant haeme moiety into equimolar quantities of biliverdin-IX*α*, carbon monoxide (CO), and ferrous iron (Fe^2+^). Biliverdin-IX*α* can then be converted into the antioxidant bilirubin, further protecting cells from oxidative insult [59, 89]. On the other hand, simultaneous production of CO and Fe^2+^ as by-products of the reaction may impact *β*-cell insulin secretion [59, 89]. While CO gas has been suggested to positively regulate insulin secretion in intact mouse islets through mobilisation of cAMP and cGMP, increased iron load has been suggested to be a key participant in DM and was associated with impaired insulin elimination from the liver, reduced insulin secretion, and attenuated insulin action and glucose transport in adipose [59]. The full role of iron overload is beyond the scope of this review, but excessive ferrous iron (Fe^2+^) produced against the backdrop of chronically high nutrient levels, increasing metabolism and accordingly elevating ROS/RNS, may lead to more Fenton reactions occurring as outlined above and consequently increased formation of hydroxyl anions. Therefore, further generation of oxidative stress in this manner may lead to enhanced induction of HO-1 expression, perpetuating a vicious cycle of ROS production in the *β*-cell.

Taken together, it is clear that excessive, uncontrolled oxidative stress contributes to pancreatic *β*-cell dysfunction, and therefore research continues to investigate the precise role of antioxidant agents in mediating *β*-cell insulin secretion and survival with a view to become potential treatments for DM, particularly in relation to GPx mimetic (e.g., ebselen) [90]. However, oxidative, nitrosative, and ER stress also play critical roles in insulin target tissues, such as skeletal muscle and adipose tissue. Consequently, the oxidative mechanisms that contribute to dysfunction in these tissues are important.

### 3.3. Inflammation-Elicited *β*-Cell Dysfunction

Proinflammatory cytokines play an important role in *β*-cell pathophysiology. The involvement of TNF-*α*, IFN-*γ*, and IL-1*β* released by infiltrating mononuclear cells in Type 1 diabetes (T1DM) associated insulitis and *β*-cell apoptosis has been documented (reviewed by [91]). Several studies have shown that IL-1*β* alone, or in combination with TNF*α* and IFN-*γ*, promotes *β*-cell dysfunction and death [92–94]. These early studies only referred to inflammation as an important player in T1DM, but not T2DM, since chronic low-grade inflammation was only later discovered to take place in T2DM [95, 96]. Interestingly, high glucose and NEFA promote intraislet production of IL-1*β* [97, 98], suggesting that cytokine-induced *β*-cell apoptosis could also occur in the metabolic syndrome and T2DM milieu.

A hallmark of insulin resistance is the deposition of amyloid in the islets and this has been linked to the activation of NLRP3 inflammasome in local macrophages, triggering IL-1*β* processing and release [99]. In this scenario, local release of IL-1*β* could autoinduce its own production through autocrine stimulation and further attract additional immune cells into the islet microenvironment. Indeed, macrophage infiltration increases along with the progression of T2DM in patients and animal models [100]. A recent study shows that the saturated fatty acid palmitate is able to activate the innate immunity via TLR4 and/or myeloid differentiation primary response gene 88 (MyD88) pathway in islets, promoting macrophage infiltration and unresolved inflammation [101]. Thus, similar signalling through cytokine receptors and consequent activation of proinflammatory transcription factors such as NF-*κ*B and the signal transducer and activator of transcription-1 (STAT-1) occur in *β*-cells, which are ultimately proapoptotic. However, such hypothesis for *β*-cell pathophysiology is still under debate. For instance, IL-1*β* is able to promote NF-*κ*B-dependent stimulation of the inducible nitric oxide synthase (iNOS, encoded by NOS2 gene) expression, contributing to cell death and dysfunction; however, the same effect was not observed in rodent *β*-cells exposed to high glucose. In addition, exposure of rat or human islets to high glucose did not increase IL-1*β* mRNA expression or NF-*κ*B DNA-binding activity [102, 103].

Undoubtedly, *β*-cell death promoted by the exposure of proinflammatory cytokines or glucolipotoxicity occurs through the intrinsic mitochondrial pathway of apoptosis. This pathway is tightly controlled by members of the B-cell lymphoma (Bcl) 2 protein family, including antiapoptotic (Bcl-2, Bcl-XL, Bcl-w, Mcl-1, and BCL2A1) and proapoptotic (Bax, Bak, DP5, PUMA, Bim, Bid, Bad, and Noxa) members. Briefly, mitochondrial apoptosis is initiated by Bax translocation and oligomerization with Bak on the mitochondrial outer membrane. This causes mitochondrial outer membrane permeabilization (MOMP) and cytochrome c release. In the cytoplasm, cytochrome c oligomerises with apoptotic protease activating factor-1 (APAF-1) to form the apoptosome, which in turn activates the initiator caspase 9. The later cleaves and activates caspases 3 and 7, which target several substrates and execute the apoptotic program [104]. Under healthy conditions, the antiapoptotic members of the Bcl-2 family bind to and inhibit Bax and Bak oligomerization. Upon activation, BH3-only proapoptotic proteins (DP5, PUMA, Bim, Bid, Bad, and Noxa) promote apoptosis either by binding and inactivation of antiapoptotic members or by direct stimulation of Bax and Bak oligomerization [105].

In unstimulated conditions, NF-*κ*B is present in an inactive form in the cytoplasm, bound to inhibitory *κ*B (I*κ*B) proteins. Following IL-1R stimulation, I*κ*B is phosphorylated at two serine residues (Ser^32^ and Ser^36^) by an I*κ*B kinase complex (IKK). These particular phosphorylation events target I*κ*B to degradation by the ubiquitin-proteasome system (UPS), releasing NF-*κ*B to translocate into the nucleus, and bind target genes promoter regions [106]. Activation of NF-*κ*B in the *β*-cell, through IL-1R signalling, regulates several genes affected by cytokines [107], including iNOS and anti- and proapoptotic members of the Bcl-2 family [104]. NF-*κ*B-dependent iNOS expression promotes nitric oxide (NO) production and the latter is involved in cytokine-induced *β*-cell secretory dysfunction and apoptosis [108]. In addition, NF-*κ*B upregulates PUMA, which activates Bax and Bak oligomerization and contributes to cytokine-induced *β*-cell apoptosis [109].

On the other hand, NF-*κ*B also induces the antiapoptotic gene BCL2A1 and the mitochondrial antioxidant Sod2 [110, 111]. Thus, NF-*κ*B-dependent gene expression changes are not purely proapoptotic; however, if sustained (e.g., under chronic low-grade inflammation), and in conjunction with additional cytokine-mediated signalling, proapoptotic signalling prevails over *β*-cell defensive systems. One such additional cytokine-mediated signalling is the JNK/c-Jun stress kinase cascade. Signalling through this particular pathway promotes degradation of the prosurvival Mcl-1 [112, 113] and upregulation of the BH3-only sensitiser DP5 [114]. NO production also exerts its proapoptotic effects in *β*-cells through activation of JNK pathway and inhibition of the kinase cascade alleviates NO *β*-cell toxicity [108]. Thus, it is the combination of chronic inflammation and glucolipotoxic environment of T2DM that causes *β*-cell failure and loss in advanced stages of the disease.

### 3.4. The Role of Endoplasmic Reticulum (ER) Stress

*β*-cell dysfunction and death are processes that are also connected to various levels of ER stress [115], particularly because hyperglycaemia requires increased “work power” by the islets that need to produce higher amounts of protein (insulin). Indeed, several physiological and environmental conditions associated with T2DM have been shown to induce ER stress in *β*-cells. The major systemic event in T2DM, insulin resistance, imposes a requirement for higher levels of insulin. *β*-cells respond to such increased demand by increasing their cell mass and insulin secretion [116]. Conditions that require sustained high levels of insulin production, such as hyperglycaemia, can result in the accumulation of unfolded proteins in the ER, since the bulk of chaperones necessary to prevent protein misfolding does not suffice protein synthesis so that this unbalanced situation triggers ER stress and, consequently, the unfolded protein response (UPR) in *β*-cells [117]. Excess nutrients, in the form of high glucose and NEFA, also promote ER stress [118]. Lastly, the negative effects of proinflammatory cytokines and oxidative stress can be mediated by ER stress responses [119].

(Pro)insulin synthesis and folding in *β*-cells take place in the lumen of the ER. However, *β*-cells often encounter situations of ER overload, for instance, when rapid production of high amounts of insulin is needed in response to hyperglycaemia. Under such situations healthy *β*-cells can boost insulin production by more than 10-fold compared to unstimulated cells, and (pro)insulin alone can account for about 50% of the total amount of proteins synthesised by the cell [120]. This can exceed the cellular folding capacity resulting in accumulation of unfolded (pro)insulin in the ER lumen, leading to ER stress [121].

In order to alleviate ER stress, cells start the biochemical program of UPR. UPR is a genetically and biochemically controlled process that is initiated within the cells in order to counteract an imbalance in ER homeostasis. It includes transcription activation of proteins involved in ER-associated protein folding and degradation, as well as temporary attenuation of mRNA translation. This process aims at restoring ER capacity in order to handle protein processing, folding, and trafficking, reestablishing proteostasis. If this fails, apoptosis is eventually triggered [122]. Accumulation of unfolded proteins is sensed by ER transmembrane proteins, such as the protein kinase RNA-like endoplasmic reticulum kinase (PERK), inositol-requiring enzyme 1 (IRE1), and the activating transcription factor (ATF) 6, which initiate the UPR program. Normally, the ER resident chaperone binding immunoglobulin protein (BiP) prevents their aggregation. However, accumulated unfolded proteins compete to BiP binding sites, allowing ER transmembrane proteins self-aggregation and activation. Once activated, PERK phosphorylates the eukaryotic translation initiation factor 2A (eIF2*α*) at (Ser^51^), resulting in global blockade of protein synthesis and alleviation of ER overload [123] (Figure 2).

Some mRNAs, however, can bypass translation blockade by phosphorylated eIF2*α*. ATF4, a transcriptional factor of the basic leucine zipper domain (bZIP) family is preferentially translated during these conditions and initiate gene expression changes associated with UPR [124]. In a similar way, IRE1 oligomerises in the ER membrane rendering its activation. It contains an endonuclease domain that, once activated, excises an intron from the X-box binding protein-1 (XBP-1). This generates the spliced form of XBP-1, which acts as a transcriptional factor promoting the expression of genes involved with misfolded protein retrotranslocation into the cytosol for proteasome degradation as well as ER-associated protein degradation [125]. High-glucose stimulus has been shown to induce IRE1 activation both* in vivo* and* ex vivo* (in isolated rodent islets) [126, 127]. ATF6 has a different mechanism of activation as compared to PERK and IRE1. Upon BiP disassociation, it migrates to the Golgi apparatus where it is cleaved and released in the cytosol. Cleaved ATF6 translocates into the nucleus to act as a transcriptional factor that has XBP-1 as one of its targets [128].

Excessive or prolonged UPR signalling can lead *β*-cells to apoptotic cell death by various different mechanisms. Possibly the most important involves upregulation and activation of CCAAT-enhancer-binding protein homologous protein (CHOP). All three branches of ER stress sensing (PERK, IRE1, and ATF6) have been implicated in activation of CHOP transcription. However, CHOP mRNA is also regulated at the translational and posttranslational levels. PERK-dependent eIF2*α* phosphorylation is essential to promote CHOP mRNA translation in a cap-independent manner (for review see [129]), similar to ATF4. Furthermore, CHOP protein is only fully activated by phosphorylation at Ser^78^ and Ser^81^ by the stress kinase P38 MAPK [130]. Once activated, CHOP exerts its effects in the nucleus where it regulates several apoptotic genes. In particular, CHOP downregulates Bcl-2 expression, tilting the balance towards a proapoptotic phenotype [131].

### 3.5. *β*-Cell Fate in UPR: Apoptosis* versus* Chronic Inflammation

As stated before, in addition to high insulin production demand, other physiological hallmarks of T2DM have been shown to trigger ER stress and *β*-cell dysfunction. Exposure to NEFA, for instance, can activate all three sensor branches of UPR. Furthermore, overexpression of BiP alleviates palmitate induced apoptosis in the rodent *β*-cell line MIN6, suggesting that saturated NEFA-induced *β*-cell death is, at least partially, mediated by ER stress [132]. A number of different mechanisms have been proposed to mediate lipotoxic-induced ER stress in *β*-cells. Palmitate promotes rapid ER calcium depletion and this was linked to *β*-cell ER stress [133]. Global proteomics analysis revealed that Carboxypeptidase E is rapidly degraded in response to *β*-cell exposure to palmitate and its degradation both precedes and is necessary for activation of ER stress response [134]. *β*-cell lipotoxicity has also been implicated in reduction of ER to Golgi protein traffic, ER overload, and consequent ER stress [135]. Mechanistically, alterations in sphingolipid metabolism were implicated in such defective protein trafficking and inhibition of ceramide synthesis reduced both ER stress and apoptosis in palmitate treated MIN6 cells [136].

Proinflammatory cytokines, especially IL-1*β*, can also induce ER stress and UPR activation in *β*-cells. The ability of this cytokine to promote NF-*κ*B-dependent iNOS expression and NO production has already been mentioned in this review. A link between cytokine-induced *β*-cell death and ER stress was established when NO production was shown to cause ER calcium depletion and ER stress [137]. The mechanism was later shown to involve NO-dependent sarcoendoplasmic reticulum pump Ca^2+^ ATPase 2b (SERCA2b) downregulation and consequent ER inability to maintain Ca^2+^ gradient between ER and cytosol, important for ER folding capacity and homeostasis [138]. Several reports demonstrate that cytokine signalling activates UPR and ER stress in *β*-cells. Strikingly, cytokine-induced NF-*κ*B activation and *β*-cell death were shown to be, at least partially, mediated by UPR and CHOP upregulation. In this scenario, CHOP depletion by siRNA prevented full activation of NF-*κ*B and its target proapoptotic genes by cytokines, confirming that the deleterious effects of proinflammatory cytokines on *β*-cells depend on the activation of the UPR program [139]. A reverse link between *β*-cell ER stress and inflammation has also been recently found. Thioredoxin-interacting protein (TXNIP), a gene induced by ER stress through the PERK and IRE1 pathways, activates the NLRP3 inflammasome and IL-1*β* production in human islets, suggesting that the crosstalk between ER stress and inflammation is a two-way process [140]. Thus, ER stress is both cause and consequence of chronic *β*-cell inflammation and these two processes are intimately interconnected. Current and future research in the area will provide further insights into mechanisms of *β*-cell dysfunction and death and so contribute to the development of future T1DM and T2DM treatments.

## 4. Conclusion

DM continues to increase in frequency, presenting a challenge to health care systems worldwide. Oxidative stress and inflammation provoked by excessive nutrient availability and obesity-induced alterations in proteostasis are key factors associated with DM progression both for the peripheral tissues and for *β*-cells within the islets. Using current molecular tools and related approaches, new studies are advancing the understanding of the dysfunctional molecular events in T2DM and may lead to better therapeutic strategies. Inflammation, ROS generation and regulation of intracellular signalling are required for homeostasis and survival. However, excess nutrient availability and low levels of physical activity lead to an imbalance in metabolic regulation linking aberrant responses of insulin secretion and action to DM progression and its severe consequences for whole body metabolism and health outcomes.
